# Distress, loneliness, and mental health during the COVID‐19 pandemic: Test of the extension of the Evolutionary Theory of Loneliness

**DOI:** 10.1111/aphw.12352

**Published:** 2022-03-09

**Authors:** Franziska Maria Keller, Christina Derksen, Lukas Kötting, Alina Dahmen, Sonia Lippke

**Affiliations:** ^1^ Jacobs University Bremen gGmbH Bremen Germany; ^2^ Dr. Becker Klinikgruppe Köln Germany; ^3^ Klinikum Wolfsburg Wolfsburg Germany

**Keywords:** anxiety, depressive symptoms, psychosomatic rehabilitation patients, serial mediation

## Abstract

COVID‐19 restrictions such as lockdowns or quarantines may increase the risk for social isolation and perceived loneliness. The mechanisms can be modeled by Cacioppo's Evolutionary Theory of Loneliness (ETL), which predicts that a lack of perceived social connectedness may lead, in the long‐term, to mental and physical health consequences. However, the association between COVID‐19 pandemic distress, mental health, and loneliness is not sufficiently understood. The present longitudinal study examined the relationship between distress and depression, and the mediating effects of anxiety and loneliness in a German rehabilitation sample (*N* = 403) at two timepoints (≤6 weeks pre‐rehabilitation; ≥12 weeks post‐rehabilitation; mean time between T1 and T2 was  52 days). Change scores between T1 and T2 were examined for the variables COVID‐19 Peritraumatic Distress Index (CPDI), anxiety, loneliness, and depression. The results of the serial mediation analysis indicated that anxiety and loneliness were able to explain the relationship between distress and depression with 42% of variance in depression accounted for. Findings extend research on the relationship between COVID‐19 and mental health by considering anxiety and loneliness as sustaining factors of depressive symptoms, thus, successfully applying the ETL. Results stress the necessity to consider anxiety and loneliness in the treatment or prevention of depression.

## INTRODUCTION

The effects of the corona virus disease 2019 (COVID‐19), which has been termed a pandemic by the World Health Organization on March 11, 2020, have led to long‐lasting and profound changes in human interaction, human health behavior, and mental well‐being. As part of the containment measures proposed by governments such as the one in Germany, individuals were, on the one hand, encouraged to reduce physical contacts as much as possible but, on the other hand, also decided to reduce their contacts to take responsibility for their own health and the health of others. Despite these regulations being effective, changes in social interaction have led to increased distress and perceived loneliness as a consequence of physical isolation as well as a deterioration in mental health displayed by higher reported symptoms of depression and anxiety (Balkhi et al., 2020). Therefore, studies examining consequences of previous virus outbreaks have concluded that distress associated with a possible infection, loneliness, boredom, symptoms of anxiety, and depression, or even suicidal thoughts may be the results of pandemic contexts and can lead to negative long‐term psychological effects or to a deterioration of a pre‐existing poor mental health status (Asmundson et al., 2020; Hao et al., 2020).

This has been especially pronounced for individuals with a prior mental health diagnosis such as individuals from psychosomatic rehabilitation clinics. Evidence has highlighted that patients with pre‐existing mental health disorders have shown to be more vulnerable to stress‐related events (Phillips et al., 2009). As those individuals need to invest more resources to cope with the original mental health disorder, they are less likely, compared to healthy individuals or the general population, to engage in active problem‐solving strategies in the face of stressful and distressing life events (Phillips et al., 2009). Several studies have evaluated the impact of the COVID‐19 pandemic as a distressing and traumatic event on the mental health status of individuals with a pre‐existing mental health disorder and of the general population. Individuals with a pre‐existing mental health condition reported increased distress, anxiety, loneliness, and depression (Groarke et al., 2020; Hao et al., 2020). As people with a pre‐existing mental health disorder are frequently neglected (Hao et al., 2020), we aim to gain a better understanding of the role of symptoms of anxiety and loneliness in the relationship between COVID‐19 Peritraumatic Distress Index (CPDI) and general symptoms of depression by using the Evolutionary Theory of Loneliness (ETL) as a theoretical basis and by investigating changes in the proposed variables over time.

### Evolutionary Theory of Loneliness

The ETL by Cacioppo provides a theoretical explanation for the perception of loneliness (Cacioppo & Cacioppo, 2018). The theory assumes that feelings of loneliness emerge, and are maintained, over time and that loneliness can affect physiological and mental health. According to the ETL, social isolation or loneliness has been termed as a signaling function that is similar to physical pain. Cacioppo's ETL states that people have an innate desire to connect to others in order to obtain and provide protection against a possible threat. If that need for social connection cannot be fulfilled, individuals may consequently report feeling lonely (Cacioppo & Hawkley, 2009).

If individuals become socially isolated, they are deprived of social connectedness especially in a pandemic context. This is the case even when individuals willingly reduce their social contacts such as in the event of the COVID‐19 pandemic. Nevertheless, seeing others as a potential threat of infection and the need to further isolate oneself can lead to maladaptive strategies such as increased avoidance behavior. As a result, lonely individuals perceive social interactions more negatively, which is reinforced by pandemic containment measures. In addition, daily routines have changed to reduce physical contact by working from home and home schooling where necessary. This might have a long‐term impact in Germany and other industrialized countries, creating a more flexible work life. Nevertheless, this flexibility and uncertainty is likely to challenge individuals especially if resources, such as stable work and good mental and physical health, are lacking. Thus, ways need to be found to reduce loneliness and fear of social interaction without endangering the containment of COVID‐19. Otherwise, this process may lead to a self‐defeating social behavior and ultimately to a vicious circle, which has the potential to cause a decreased mental and physical health status.

Mushtaq et al. (2014) have shown that increased loneliness has been associated with a decreased mental health status. The relationship between anxiety and loneliness, as well as between loneliness and depression, has been well studied with respect to the general population. However, adequate research is lacking for psychosomatic rehabilitation patients with a pre‐existing mental health diagnosis. Therefore, with respect to the current study, experienced peritraumatic distress may possess the function of signaling potential danger that may lead to feelings of distress. These feelings, in turn, have the potential to increase anxiety‐related symptoms leading to a voluntary withdrawal from social situations and connections as a safety measure. If the social withdrawal is maintained for a longer period of time, it likely results in feelings of loneliness. This may, in turn, result in increased depressive symptoms.

Depression is characterized by lack of interest, general withdrawal, feelings of worthlessness, loneliness, or reduced interest and pleasure in activities. Hence, with regard to the ETL, it may be assumed that specifically during the COVID‐19 pandemic the interplay between peritraumatic distress, anxiety, loneliness, and depression may depict a vicious circle as one part of the ETL which will be examined further in the present study.

### CPDI in association with symptoms of depression

Peritraumatic distress, as conceptualized by Brunet et al. (2001), describes emotional and physical responses either at the time, or immediately after, a traumatic event. It has been termed as a state condition with decreasing or, even diminishing, perceived severity over time. The COVID‐19 pandemic represents a traumatic series of events over a prolonged time that may lead to peritraumatic distress accompanied by psychiatric outcomes such as depression, anxiety, acute stress disorder, sleep disturbances, traumatic grief disorders, or psychological distress (Megalakaki et al., 2021). Liu et al. (2021) have shown that the psychological distress due to the COVID‐19 pandemic has significantly increased from 24% to 66% between April 2020 and May to September 2020 in Germany. To measure peritraumatic distress, Brunet et al. (2001) originally developed the peritraumatic distress inventory that was used as the basis for the development of the COVID‐19 CPDI. The CPDI assesses several aspects related to the psychological and physiological impact of the COVID‐19 pandemic (Qiu et al., 2020). In addition, several studies have examined the association between general peritraumatic distress and depression or post‐disaster depression (Bell et al., 2017). However, Megalakaki et al. (2021) were the first to examine the association between CPDI and mental health conditions in the context of the COVID‐19 pandemic. Their results underline the assumption that an increased CPDI index is associated with increased symptoms related to depression and are in line with previous research on general peritraumatic distress and depression, respectively.

### CPDI and the association with anxiety and loneliness

The definition of an anxiety syndrome is attributed to an increase of pandemic‐related psychological distress, fear, and generalized stress (Bäuerle et al., 2020) in connection with the COVID‐19 pandemic. The anxiety syndrome according to Nikčević and Spada (2020) has been characterized by increased avoidance, checking, worrying, and threat monitoring, which have all been noticed during the COVID‐19 pandemic. Results from the beginning of the COVID‐19 pandemic have reported increased stress‐related and anxiety‐related symptoms as a response to the pandemic (Wang, Pan, et al., 2020). Literature has also highlighted the association between pandemic‐related psychological distress and anxiety as those suffering from increased psychological distress also reported higher levels of anxiety (Wheaton et al., 2012). Specifically, with regard to COVID‐19 peritraumatic distress, a higher CPDI has been able to predict symptoms of anxiety (Megalakaki et al., 2021). However, the role of loneliness in this context has, so far, not been researched.

Loneliness has been defined as “a distressing feeling that accompanies the perception that one's social needs are not being met by the quantity or especially the quality of one's social relationships” (Hawkley & Cacioppo, 2010, page 1). With regard to the association between CPDI and loneliness, literature revealed that increased peritraumatic distress corresponds with living alone and the subjective perception of being avoided by others or the social environment (Liu & Heinz, 2020). These results are in line with research by Liu and Heinz (2020) suggesting that increased psychological distress, such as the distress experienced during the COVID‐19 pandemic, has consequently been associated with an increase in loneliness. However, due to virus containment measures individuals are not aiming to become lonely but only aim for social isolation to comply with legal requirements and to prevent the spread of the SARS‐CoV‐2 virus. Hence, loneliness and social isolation need to be treated as distinct concepts.

Results have highlighted that individuals with an increased generalized anxiety score reported increased symptoms of loneliness. Alasmawi et al. (2020) have shown that perceived loneliness or the severity of reported loneliness differs depending on the mental health diagnosis. Hence, individuals with a common mental health disorder (i.e. anxiety) and people with a personality disorder reported higher rates of loneliness compared to individuals diagnosed with a psychotic disorder.

### Anxiety and loneliness in association with symptoms of depression

Symptoms of anxiety and loneliness are related to symptoms of depression in psychosomatic rehabilitation patients. Protective measures such as lockdowns or quarantining have been known to have a deteriorating effect on mental health (Brooks et al., 2020). In ambiguous and uncertain situations (such as a pandemic) insecurity, fear, and distress among the population may increase (Liu et al., 2020, 2021; Mamun et al., 2021). Consequently, increased intensity of fear and distress may lead to increased worrying about becoming infected (Lin, 2020). These negative emotions associated with worrying and fear can have detrimental effects on well‐being and have the potential to evolve into or intensify severe psychological illnesses such as depression, anxiety, and in extreme situations, even suicidal thoughts (Mamun et al., 2021). Therefore, fears associated with an infection may lead to chronic vigilance for COVID‐19‐related stressors that may contribute to a development of or an increase in the symptomatology of anxiety (Harding et al., 2008).

Previous research has highlighted the strong association between loneliness and anxiety and has shown that increased loneliness was associated with a higher score in affective symptoms related to an affective disorder such as depression (Wang, Lloyd‐Evans, et al., 2020). Individuals with a lower perceived social support or social connectedness, as during the COVID‐19 pandemic, display increased levels of loneliness. This can, in turn, lead to negative cognitive biases which consequently reinforce and foster feelings and associated behaviors related to depression (West et al., 1986). Additionally, results by McPherson et al. (2021) revealed that loneliness experienced at the beginning of a lockdown provided a risk factor for developing clinically significant depression. Gallagher et al. (2021) have examined whether feelings of loneliness have the potential to increase or exacerbate the risk of depression in individuals with a pre‐existing cancer diagnosis during COVID‐19. Results highlighted that loneliness experienced during the pandemic was predictive of an increased risk of depression.

While fleeting perceptions of loneliness can lead to adaptive responses such as an active search for connectedness, chronic loneliness can lead to maladaptive strategies such as negative cognitions and hence further social withdrawal (Hawkley & Cacioppo, 2010). The COVID‐19 pandemic has the potential to enhance this negative “feedback loop” since it reinforces withdrawal and distancing. This is especially true for individuals with limited health since they might lack strategies or opportunities for compensation (Greig et al., 2021). The ELT (Cacioppo & Cacioppo, 2018) can further explain the mechanisms. Nevertheless, it has rarely been applied to individuals with mental or physical health impairments.

### The current study

The aim of the current study is to investigate and examine symptoms of anxiety and loneliness as serial mediators in the positive relationship between CPDI and symptoms of depression in a sample of psychosomatic rehabilitation patients. This study answers the research question: How are anxiety and loneliness associated with the relationship between peritraumatic stress and symptoms of depression? Consequently, we hypothesize that higher CPDI predicts higher symptoms of depression through a serial mediation pathway from higher symptoms of anxiety and loneliness in a longitudinal manner.

## METHODS

The present study was funded as part of the research project “TeamBaby – Communication and patient safety in gynecology and obstetrics” (ClinicalTrials.gov Identifier: NCT03855735), which is funded by the Innovation Fund of the Federal Joint Committee (Project No. 01VSF18023). In addition, this stuy was funded by the research project “ASAP ‐ Assisted Immediate Augmented Post‐/Long‐COVID Plan” (ClinicalTrials.gov Identifier: NCT05238415), which is supported by the Bayerisches Landesamt für Gesundheit und Lebensmittelsicherheit (Project No. AZ‐2490‐PC‐2021‐V7‐D56613/2021). The current study and data collection was conducted as part of the project “Anhand‐COVID19‐Offer to achieve treatment and rehabilitation goals in compliance with hygiene and social‐distancing rules” (ClinicalTrials.gov Identifier: NCT04453475), which is supported by the Dr. Becker clinic group.

### Recruitment of the psychosomatic rehabilitation patients

Participants were recruited through four participating medical rehabilitation clinics from the Dr. Becker clinic group. Patients were admitted to the clinics due to psychosomatic diagnoses and were invited to take part in the online survey through their clinic before starting their treatment. Before participation, patients were informed about the study by writing on the rehabilitation clinic group's online portal. The survey was administered via the survey platform Unipark.

All data collected as part of this study were pseudonymized. No form of compensation was offered to participants. The survey was administered between April 2021 and September 2021. Patients were asked to fill out the online questionnaire from 6 weeks before until the first day of rehabilitation (T1) as well as after their rehabilitation stay (T2), which was possible up to 12 weeks post rehabilitation. At baseline, *N* = 676 participants participated in the study. After rehabilitation, a total of 273 participants dropped out, leaving 403 participants who completed the survey at both measurement timepoints.

Due to the pandemic situation, contact restrictions were introduced (i.e. no visitors allowed or only one or two visitors at a fixed time, reduced group sizes for therapy sessions and the avoidance of physical contact) to reduce the potential of a COVID‐19 infection. These contact restrictions persisted during the entire data collection phase and were applicable for all patients.^1^ Ethical approval for the online survey concerning psychosomatic rehabilitation patients was given by the Ethics Committee at Jacobs University Bremen (protocol code 2020_09) on June 25, 2020.

### Participants from the psychosomatic rehabilitation clinics

Of the 403 patients participating at both measurement timepoints, 264 (65.8%) patients were female. Participants' age ranged between 18 to above 60 years. Forty‐nine (12.2%) were 39 years or younger, 84 (20.9%) patients between 40 and 49, 205 (51.0%) between 50 and 59 years of age, and 64 patients (15.9%) were 60 years or older. Educational level was categorized into four groups: 67 (16.9%) patients indicated to have received 10 or 11 years of schooling, 77 (19.4%) answered to have received 12 or more years of schooling, 185 (46.7%) indicated to have obtained vocational training, and 67 (16.9%) indicated to have obtained a university degree.

Age, gender, and educational levels were measured as categorical variables. The 232 (61.2%) patients were diagnosed upon discharge with a mood (affective) disorder, 135 (35.6%) were diagnosed with a neurotic, stress‐related, and somatoform disorder, and 12 (3.2%) patients were given a diagnosis pertaining to other diagnoses. The 84 participants answered to the current status of their living situation. Eighteen (21.4%) indicted to be living alone, and 66 (78.6%) answered to be living with at least one other person in a shared household.^2^ Patients spend on average 52 days (SD = 12 days) in the clinics. The minimum and maximum of days spend at the rehabilitation clinics were 2 days and 97 days, respectively. On average 67 days passed between taking part in survey at T1 and at T2 (SD = 24 days) with a minimum of 26 and a maximum of 209 days.

### Instruments

#### COVID‐19 CPDI questionnaire

The COVID‐19 CPDI was assessed by 24 items at T1 and T2 (Qiu et al., 2020). All items were measured on a 5‐point Likert scale from 0 (*not at all*) to 4 (*extremely*). The sum of all items results in a raw score. The display score can be obtained by adding 4 to the raw score. The total score of the CPDI ranges from 0 to 100. All items were aggregated in terms of a sum score with a good internal reliability with Cronbach's alpha of 0.87 at T1 and Cronbach's alpha of 0.90 at T2.

#### Mental health—Symptoms of depression and anxiety

Mental Health was measured by symptoms of depression and anxiety via the 2‐item Patient Health Questionnaire (PHQ‐2; Kroenke et al., 2003), and the 2‐item Generalized Anxiety Disorder scale (GAD‐2; Kroenke et al., 2007). The PHQ‐2 (Kroenke et al., 2003) assesses symptoms related to depression, and the GAD‐2 (Kroenke et al., 2007) measures symptoms of anxiety. Symptoms of depression were measured by the following: “Over the last 2 weeks, how often have you been bothered by any of the following problems? Little interest or pleasure in doing things” and “Feeling down, depressed or hopeless.” Anxiety‐related symptoms were measured by the two items: “Over the last 2 weeks, how often have you been bothered by any of the following problems? Feeling nervous, anxious or on edge” and “Not being able to stop or control worrying.” All four items were rated on a 4‐point Likert scale from 0 (*not at all*) to 4 (*nearly every day*). Summed scores of 3 or higher indicated a probable case of a depression or an anxiety diagnosis. With regard to the current study, the PHQ‐2 (internal reliability with Spearman's *ρ* = 0.71 at T1 and with Spearman's *ρ* = 0.75 at T2) and the GAD‐2 (internal reliability with Spearman's *ρ* = 0.71 at T1 with Spearman's *ρ* = 0.76 at T2) were not used as a diagnostic tool, but rather were used as an indicator of symptom intensity.

### Loneliness

Perceived loneliness was assessed by the item: “How often do you feel unhappy to be alone?” from the UCLA Loneliness Scale (Russell, 1996). In addition, the item “How often do you feel lonely?” stemming from the Center for Epidemiologic Studies‐Depression Scale (CES‐D; Radloff, 1977) was used. Both items were measured on a 4‐point Likert scale from 1 (*not at all*) to 4 (*almost every day*). The items were aggregated in terms of a sum score which revealed good internal reliability with Spearman's *ρ* of 0.81 at T1 and Spearman's *ρ* of 0.85 at T2.

### Socio‐demographic characteristics

Data on socio‐demographic characteristics considered gender, age, educational level, and ICD‐10 diagnosis upon rehabilitation discharge. Gender was categorized into two sub‐groups: female and male. For the purpose of this study, age in years was assessed in the following four categories: <39, 40–49, 50–59, and >60. Age needed to be assessed in categories at the clinics to ensure confidentiality with regard to the patient data used as part of this study. In addition, educational level was categorized into four groups and information on the ICD‐10 diagnosis was categorized into three groups.

### Statistical analyses

For all analyses, SPSS Version 28 was used (IBM Corp., Armonk, NY, USA). For all analyses a change score between T1 and T2 for all variables at interest was calculated to analyze the longitudinal association between the variables. Therefore, scores reported at T1 were subtracted by scores reported at T2. First, the correlations of the study variables at interest (CPDI, depression, anxiety, and loneliness) were analyzed by Pearson's correlation. Afterwards, a multiple step mediation analysis was conducted to test the hypothesis. As part of this analysis, the dependent variable, that is, symptoms of depression, was regressed on the independent variable, that is, CPDI via a chain of two serial mediators (M1 and M2; i.e. anxiety and loneliness). Therefore, the independent variable was hypothesized to predict M1 in the first step. M1 was further modeled to predict M2 in a second step and finally, M2 was hypothesized to predict the dependent variable in the third and last step.

**FIGURE 1:**
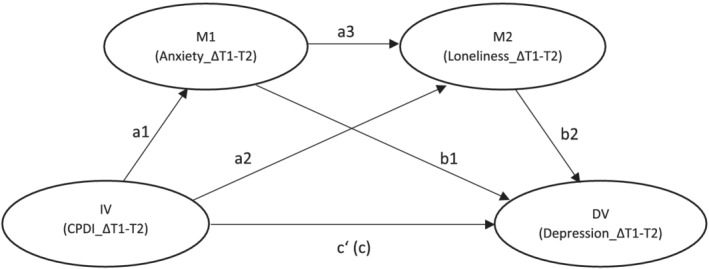
Theoretical model of the serial mediation model. Note: The serial mediation model contains six path coefficients (a1, a2, a3, b1, b2, c′) and the total effect (c). *Note*. IV = independent variable, DV = dependent variable, M1 = mediator 1, M2 = mediator 2

The serial mediation model was analyzed using the PROCESS macro Model 6 for SPSS (Hayes, 2013). The bias‐corrected 95% confidence interval (CI) was calculated with 5000 bootstrapping re‐samples. If the value zero was not included in the 95% CI, it was indicated that the mediating effect was significant. A statistical significance was defined as a two‐tailed value of *p* < .05. The analysis of the serial mediation was controlled for the covariates age, gender, educational level, and ICD‐10 diagnosis upon discharge. We assessed multicollinearity by using the variance inflation factor (VIF) test. The VIF as well as the tolerance values indicated no problems with multicollinearity as all values for VIF were <10 (Hair et al., 2014). The theoretical model is depicted in Figure 1.

### Data availability

The data presented in this study are available on request from the corresponding author. The data are not publicly available due to confidential patient information being used.

## RESULTS

### Attrition analysis

As part of the attrition analysis we examined differences between those who retained versus those who dropped out after measurement point T1 concerning the four variables at interest. Hence, the results highlight no differences between the patients who dropped out after measurement timepoint T1 and those who retained (see Appendix A). To validate the findings of the present study, a validation study was performed with all *n* = 676 patients. Missing data of patients who dropped out from the study after T1 was imputed by means of the expectation–maximization‐algorithm (see Appendix B). Results between the original study and the validated study with imputed data did not differ with respect to the serial mediation model.

### Bivariate correlations among all variables

Table C1 provides an overview over means (M), standard deviations (*SD*), and bivariate Pearson's correlations (*r*) among all examined study variables at both measurement timepoints T1 and T2. The results of the correlation analysis were consistent with our hypothesis as all variables measuring change from pre‐ to post‐rehabilitation were significantly associated with one and another at the level of *p* < .01 (two‐tailed). In addition, intercorrelations between all variables at T1, T2, and change scores are represented in Table C1.

### Longitudinal serial mediation analysis

**FIGURE 2:**
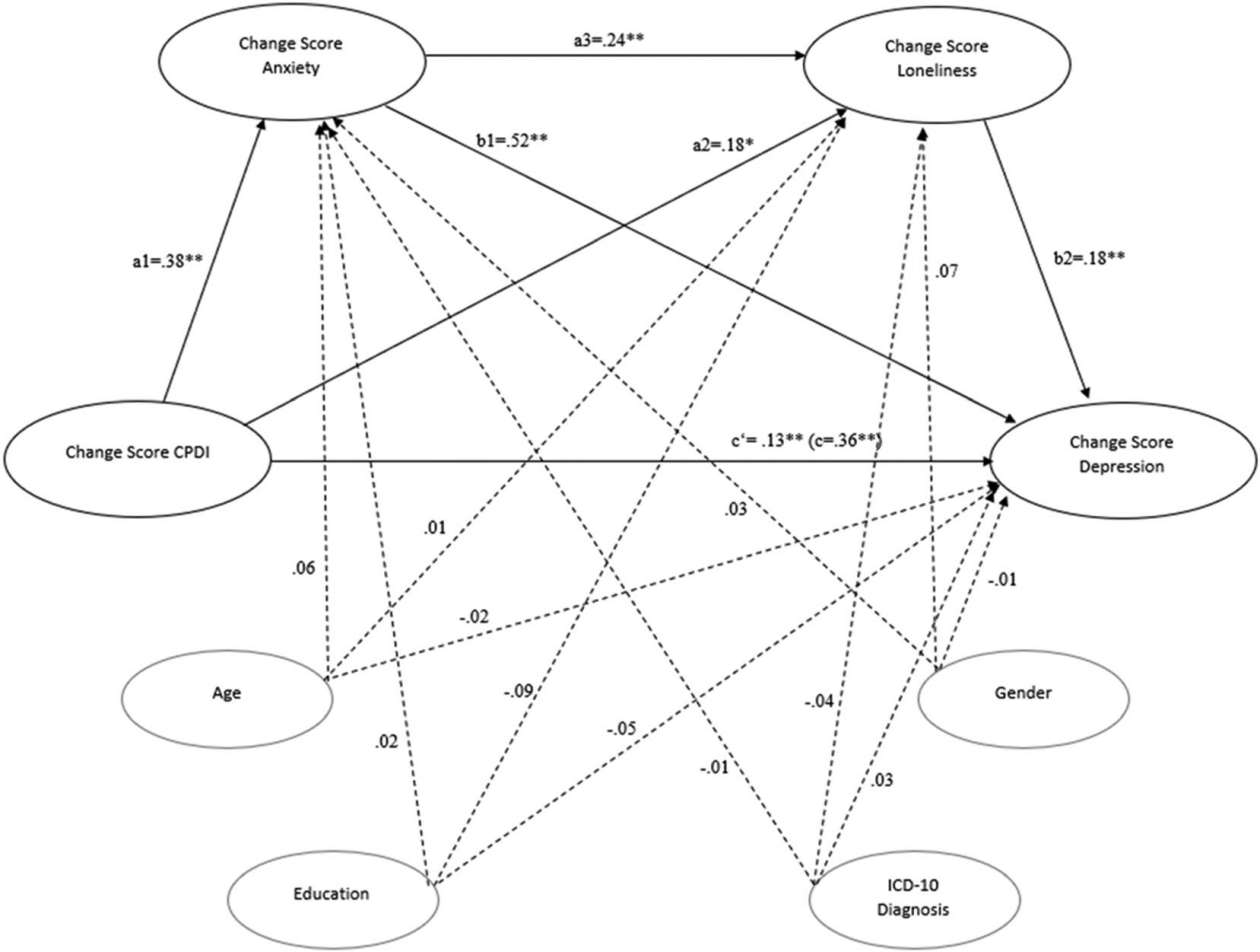
Longitudinal serial mediation model for COVID‐19 Peritraumatic Distress Index (CPDI), symptoms of anxiety, loneliness, and symptoms of depression in *N* = 342 rehabilitation patients. *Note*. The model is controlled for age, gender, education, and ICD‐10 diagnosis. Reported coefficients are standardized betas coefficients, in brackets is the total effect; * *p* < .05; ** *p* < .01 (see Figure B2 for the full sample)

The results of the longitudinal serial mediation analysis for CPDI (IV) on anxiety (M1) and loneliness (M2) on symptoms of depression (DV) controlling for age, gender, educational level, and ICD‐10 diagnosis are shown in Figure 2 and Table 1. The change score of the CPDI was significantly positively associated with the change score of symptoms of generalized anxiety. The change score of symptoms of generalized anxiety was in turn positively associated with the change score of loneliness. Further, a significant association was found between the change score of the CPDI index and the change score of loneliness. Finally, the change score of loneliness significantly predicted the change score of symptoms of depression. The total effect of the independent variable CPDI on the dependent variable symptoms of depression was significant and also remained significant upon the inclusion of the mediator variables in the model.

**TABLE 1:** Hypothesized longitudinal serial mediation model of symptoms of anxiety and loneliness between CPDI and symptoms of depression in *N* = 342 rehabilitation patients

Pathway	Effect	SE	BootLLCI	BootULCI
Total effect (c)	0.364	0.008	0.041	0.072
Direct effect (c′)	0.134	0.007	0.007	0.035
a1	0.381	0.009	0.043	0.076
a2	0.111	0.009	0.002	0.036
a3	0.241	0.062	0.116	0.358
b1	0.507	0.051	0.408	0.606
b2	0.184	0.045	0.099	0.274
Indirect effects				
Total indirect effects	0.230	0.035	0.163	0.299
Indirect 1	0.193	0.032	0.134	0.257
Indirect 2	0.021	0.013	0.002	0.048
Indirect 3	0.017	0.008	0.006	0.031

*Abbreviations*: Indirect 1, CPDI → symptoms of anxiety → symptoms of depression; Indirect 2, CPDI→ loneliness → symptoms of depression; Indirect 3, CPDI→ symptoms of anxiety → loneliness → symptoms of loneliness. BootLLCI, bootstrapping lower limit confidence interval; BootULCI, bootstrapping upper limit confidence interval; SE, standard error; Effect, standardized regression coefficient (see Table B2 for the full sample).

With regard to the present serial mediation path model, three possible indirect effects were examined. The total indirect path from CPDI to symptoms of depression through the mediator variables, symptoms of anxiety and loneliness, was significant. In addition, the specific indirect path through symptoms of anxiety was also significant (*ß* = .173, 95% CI [0.106, 0.243]) as well as the specific indirect path through loneliness (*ß* = .176, 95% CI [0.118, 0.241]).

Symptoms of anxiety as well as loneliness served as independent mediators of the relationship between CPDI and symptoms of depression. All covariates (gender, age, educational level, and ICD‐10 diagnosis) were not significantly associated with either variable in the serial mediation model. Overall, 42.11% of the variance in symptoms of depression was accounted for.

## DISCUSSION

The present study examined the mechanisms between distress, anxiety, loneliness, and depression based on the ETL, a potential vicious circle. Hence, this study tested whether COVID‐19 peritraumatic distress predicted higher depressive symptoms through a serial mediation pathway of increased anxiety and increased reported loneliness. The present findings support our hypothesis that symptoms of anxiety and loneliness are serial mediators in the positive association between COVID‐19 peritraumatic distress and symptoms of depression in a sample of psychosomatic rehabilitation patients. The serial mediation was conducted in a longitudinal fashion by examining change scores (before rehabilitation – after rehabilitation) for all variables. In line with the proposed hypothesis, a higher CPDI predicted symptoms of depression through a serial mediation pathway of symptoms of anxiety and loneliness. The results with regard to the direct effect suggested that peritraumatic distress is positively correlated with symptoms of depression in medical, psychosomatic rehabilitation patients, as other studies have shown before (Megalakaki et al., 2021).

Additionally, to the overall mediation effect, the mediators, symptoms of anxiety and loneliness, also served as individual mediators between peritraumatic distress (CPDI) and symptoms of depression. Hence, as no previous study has examined this specific mediation effect, the present research adds new empirical evidence to provide a greater understanding of how the relationship between peritraumatic distress and depression are connected in the pandemic context.

Further, our results are in line with a study by Megalakaki et al. (2021) revealing a positive association between an increase in COVID‐19 peritraumatic distress and increased generalized anxiety. Our data also integrate well with previous research. Individuals with a pre‐existing mental health diagnosis during the COVID‐19 pandemic displayed a positive link between peritraumatic distress and an increased anxiety score (Megalakaki et al., 2021; Wheaton et al., 2012). Individuals with higher reported distress have also been shown to display higher signs of worry and fear related to an anxiety disorder (Liu et al., 2020).

The present findings reflect previous research indicating that higher peritraumatic distress is positively associated with feelings of loneliness due to the mitigation strategies of physically distancing oneself due to the COVID‐19 pandemic. Earlier studies found that individuals with an increased peritraumatic distress reported increased loneliness, a lower frequency of social networks, and more fear of being alone in the future compared to individuals with a lower CPDI (Liu & Heinz, 2020). This was especially pronounced for individuals with a pre‐existing mental health disorder.

Our results confirm a positive association between peritraumatic distress and loneliness. This association is also in line with previous studies showing that individuals with increased distress reported a lower number of social contacts and a lower frequency of weekly contacts with others (Benke et al., 2020). Therefore, traumatic distress due to the COVID‐19 pandemic seems to be positively associated with generalized anxiety and loneliness as well.

The significant association between anxiety and loneliness demonstrates that symptoms of anxiety are associated with loneliness in the domain of symptoms of depression. The present results can be well integrated into existing literature. Previous studies found that loneliness has been significantly associated with an increase in depressive symptoms (Gallagher et al., 2021; McPherson et al., 2021). Further, it has been shown that increased anxiety has the ability to increase levels of experienced loneliness (Ebesutani et al., 2015). In addition, reported loneliness appears to precede and lead to increased reported symptoms of depression (Gallagher et al., 2021; McPherson et al., 2021). So far, only one study by Ebesutani et al. (2015) has examined the role of loneliness as a mediator in the relationship between anxiety and depression in youth. Therefore, the present findings extend the understanding of this association.

In addition to the multiple step mediation effect, present results revelated that both anxiety and loneliness fully mediated the relationship between peritraumatic distress and depression. This underlines the importance of recognizing symptoms of anxiety and loneliness as sustaining factors for depression. While, according to cognitive models of anxiety and depression, the core difference between depression and anxiety is the emotional pattern (Dobson, 1985). In other words, anxiety is future oriented and predictive of a potential threat. In contrary, depression is associated with either imminent or past events which bear a potential threat on self‐esteem. Literature has postulated that anxiety and depression often co‐occur (Borsboom, 2017). However, rather than viewing anxiety and depression as separate or categorical entities, literature has termed an alternative view called the network approach to psychopathology (Borsboom, 2017).

The network approach of psychopathology postulates that symptoms of both anxiety and depression actively reinforce one another leading to a comorbidity (Borsboom, 2017). For example, an individual perceiving a pandemic fear as threatening may experience physical and cognitive symptoms of anxiety. The individual may also be tenser when confronted with a threatening situation or anticipating possible consequences of a threatening situation, potentially developing the tendency to avoid situations in which a threat may be predominant. The consequent loss of social interactions and the associated perceived loneliness related to avoidance may lead to the development of symptoms related to a depressive episode (i.e. lower self‐esteem and lower self‐worth).

Overall, our analyses and findings were based on the ETL which suggests that individuals who feel lonelier tend to engage in self‐defeating and pessimistic cognitions. This may, in turn, increase the risk for depressive symptoms (Cacioppo & Cacioppo, 2018; Cacioppo & Hawkley, 2009). The ETL theory seems applicable to the new background of the COVID‐19 pandemic. Our results thus provide an extension to the ETL theory in that they suggest that distress associated with a traumatic or uncertain situation (such as the COVID‐19 pandemic) and a consequent increase in anxiety. This could play a role in the development and maintenance of loneliness. The ETL has been discussed with regard to disability, which could be a crucial barrier against adaptive strategies, hence facilitating the development of maladaptive strategies. Nevertheless, only very few previous approaches have been made in applying it to people suffering from psychological and physical health conditions. Hence, our study extends the applicability to psychosomatic rehabilitation patients.

### Implications

Our results indicate that reducing anxiety as well as loneliness is useful and necessary for patients with a pre‐existing psychological disorder to reduce or stabilize reported symptoms of depression. Therefore, it is of importance to provide patients adequate information about the COVID‐19 pandemic and its SARS‐COV2 virus in the media to reduce COVID‐related distress and anxiety, respectively. As recommended, a common coping strategy during the pandemic has been to limit exposure to news reports. This has been of importance especially for individuals with a pre‐existing mental health condition, as those individuals may be more sensitive to distressing information which may in turn spark an anxious and depressive reaction (Asmundson et al., 2020). This reaction may be further complicated and strained by the loss of face‐to‐face social support networks due to the requirements to physically isolate from others (Asmundson et al., 2020).

Research suggests that the loss of social networks in the face of the pandemic may consequently lead to an increase in mood‐related disorders (Grey et al., 2020). As individuals with a pre‐existing mental health condition are often overwhelmed with applying adequate and effective coping strategies, support strategies need to be put into place. Therefore, individuals with a pre‐existing reduced mental health status should receive interventions that promote resources and protective factors, such as character strength to increase individual well‐being (Umucu et al., 2021). In addition, low threshold digital or face‐to‐face support networks should be offered to individuals to reduce perceived anxiety by providing adequate and adaptive coping strategies that can be helpful in overcoming loneliness according to the ELT (Cacioppo & Cacioppo, 2018). Furthermore, individuals should be encouraged to engage in more social contact either face‐to‐face with necessary protective measures or in a digital mode to alleviate feelings of loneliness. By forming new contacts, or reactivating existing social contacts, patients may be able to reduce reported symptoms of depression. Therefore, to break through the vicious circle of fear that leads to depression, the mediating and, therefore, maintaining factors of anxiety and loneliness need to be acknowledged and treated. Since the pandemic is likely to become endemic and it is questionable whether there will be an “end of COVID‐19”, it is necessary to face the possibility of a “new normal” including different strategies to contain the virus in daily life: To support this process of acceptance and integration, activities should be taken up that satisfy basic psychological needs to enhance satisfaction, coping, and self‐regulatory skills (Behzadnia & FatahModares, 2020), thereby decreasing the potential psychological burden on mental health. In this way, individuals with a limited mental and overall health can be supported in finding their “new normal,” that is, by using digital methods of communication and work, especially for rehabilitation patients who might be challenged in adapting to a new normal.

Furthermore, the present findings suggest the necessity for tailoring COVID‐19‐related mental health interventions to support patients with a pre‐existing mental health condition to facilitate them in coping with fearful and uncertain situations effectively. In addition, our results have highlighted a significant relationship between anxiety and depression, indicated by individuals with higher symptoms of anxiety who also reported higher symptoms of depression.

### Limitations and suggestions for further research

Several study limitations need to be recognized. First, the study measured depressive and anxiety symptoms by means of two two‐item scales. To examine the extent of the symptoms of depression and anxiety, a broader assessment may be necessary. In addition, as there are currently no comparable longitudinal studies available which assess depression and anxiety at similar time points within the pandemic in the general population in Germany, the generalizability of our findings may be limited. Therefore, future studies should also focus on comparing symptoms between psychosomatic rehabilitation patients and the general population and similar points within the COVID‐19 pandemic. Further, we observed a rather high drop‐out rate during the follow‐up period of about 40.4%. Even though the high drop‐out rate might limit the generalizability of the current results, several other studies have encountered similar drop‐out rates in studies performed with psychosomatic rehabilitation patients (e.g. Lippke et al., 2021). Further, the results obtained by the validation study, which assess the serial mediation model with imputed data for measurement timepoint two, mimic the results of the present study.

Moreover, future studies should evaluate the specific mechanisms, that is, whether the different variables interrelate or whether there are real causal effects. Accordingly, experimental designs are needed to investigate this. Also, examining and analyzing longitudinal trends in symptom changes in order to draw conclusions on long‐lasting symptoms changes in psychosomatic rehabilitation patients is necessary. Moreover, as this study only examined anxiety, loneliness, and depression only within a small time frame (i.e. before and after rehabilitation), future research should also measure the constructs at additional time points after rehabilitation. Furthermore, no causal conclusion can be drawn as part of our study, as we did not adopt an experimental research design. Translating our findings into interventions integrated in the rehabilitation process is needed. Such interventions should be tested in randomized controlled trial with a waiting control group.

## CONCLUSION

Our study suggests that an increase in COVID‐19 peritraumatic distress, as well as intensified symptoms of anxiety and loneliness, may lead to elevated symptoms of depression or are, at least, maintaining factors for depression among psychosomatic rehabilitation patients. A theory that can be used in psychosomatic rehabilitation is the ETL. Therefore, this paper stresses the necessity to not only treat ICD‐10 diagnoses such as anxiety and depression, but also to acknowledge loneliness and stress associated with the COVID‐19 pandemic as a sustaining factor.

## CONFLICT OF INTEREST

The authors declare no conflict of interest.

## ETHICS STATEMENT

Ethical approval for study was given by the Ethics Committee at Jacobs University Bremen (protocol code 2020_09 and date of approval: 25 June 2020).

## CLINICAL TRIAL REGISTRATION

ClinicalTrials.gov Identifier: NCT04453475.

## Figures and Tables

**FIGURE B2:**
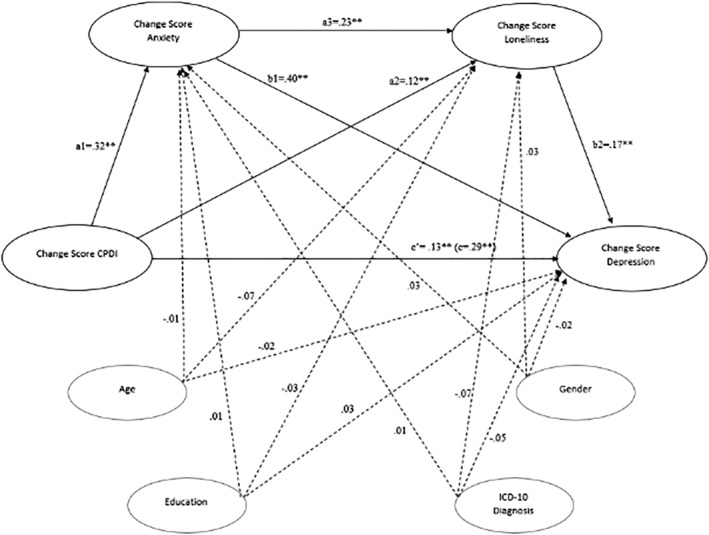
Validating the longitudinal serial mediation model COVID‐19 Peritraumatic Distress Index (CPDI), symptoms of anxiety, loneliness, and symptoms of depression with *N* = 651 rehabilitation patients (retaining dropouts by means of missing imputation). *Note*. The model is controlled for age, gender, education, ICD‐10 diagnosis. Reported coefficients are standardized betas coefficients, in brackets is the total effect; ** *p* < .01 With regard to the present serial mediation path model, three possible indirect effects were examined: (1) the total indirect path from CPDI to symptoms of depression through symptoms of anxiety and loneliness which was significant; (2) the specific indirect path through symptoms of anxiety which was not significant (*ß* = .107, 95% CI [0.063, 0.154]); (3) and the specific indirect path through loneliness (*ß* = .115, 95% CI [0.077, 0.156]), which was also significant

## Data Availability

The data presented in this study are available on request from the corresponding author. The data are not publicly available due to confidential patient data being used.

## ENDNOTES

^1^
Analyses examining differences in time‐periods (periods April to Mai 2021, June to July 2021, and August to September 2021) revealed no significant difference with regard to the variables at interest.

^2^
No significant differences with regard to loneliness were found between individuals with different living arrangements.

## APPENDIX A

### Attrition analysis

*CPDI, symptoms of depression, symptoms of anxiety, and symptoms of loneliness*. The attrition analysis revealed no significant differences with regard CPDI (*M*_Drop‐out after T1_ = 32.13; *M*_longitudinal sample_ = 31.24; *t*[998] = 1.42; *p* = .156) for symptoms of depression (*M*_Drop‐out after T1_ = 3.42; *M*_longitudinal sample_ = 3.36; *t*[998] = 0.93; *p* = .350), symptoms of anxiety (*M*_Drop‐out after T1_ = 3.43; *M*_longitudinal sample_ = 3.41; *t*[998] = 0.263; *p* = .793), and symptoms of loneliness (*M*_Drop‐out after T1_ = 4.59; *M*_longitudinal sample_ = 4.51; *t*[998] = 0.870; *p* = .384).

## APPENDIX B

### Validation of results

#### Aim

To validate the findings of the study examining the longitudinal relationship between symptoms of anxiety and loneliness as serial mediators in the relationship between CPDI and symptoms of depression, data from the entire sample, including drop‐out participants, were evaluated. To impute missing data at measurement timepoint T2 (post‐rehabilitation), the expectation–maximization‐algorithms (EM imputation) was chosen.

#### Participants from the psychosomatic rehabilitation clinics

Of the 676 recruited psychosomatic rehabilitation patients participating at both measurement time‐points, 455 (67.3%) patients were female. Participants' age ranged between 18 to above 60 years. One hundred seventeen (17.3%) were 39 years or younger, 154 (22.8%) patients between 40 and 49, 304 (45.0%) between 50 and 59 years of age, and 100 patients (14.8%) were 60 years or older. Educational level was categorized into four groups: 106 (15.9%) patients indicated to have received 10 or 11 years of schooling, 130 (19.5%) answered to have received 12 or more years of schooling, 301 (45.1%) indicated to have obtained vocational training, and 130 (19.2%) indicated to have obtained a university degree. The 124 participants answered to the current status of their living situation. Thirty‐six (29.0%) indicted to be living alone, and 88 (71.0%) answered to be living with at least one other person in a shared household.^3^ Age, gender, and educational level were measured as categorical variables. The 412 (60.9%) patients were diagnosed upon discharge with a mood (affective) disorder, 239 (35.4%) were diagnosed with a neurotic, stress‐related, and somatoform disorder, and 25 (3.7%) patients were given a diagnosis pertaining to other diagnoses.

#### Instruments

The same instruments as in the original study were used for the validation study.

#### Statistical analyses

For the validation study, SPSS Version 28 was used (IBM Corp., Armonk, NY, USA). Change scores between T1 and T2 were calculated for all variables at interest (T1 scores–T2 scores). Correlations between all variables (CPDI, anxiety, loneliness, and depression) were analyzed by Pearson correlation. A multiple step mediation analysis was conducted to validate the results of the original study. Depression, as the dependent variable, is regressed on CPDI (the independent variable) via a chain of the mediators: anxiety and loneliness.

#### Bivariate correlations among all variables

An overview of means (M) and standard deviations (*SD*), as well as of the Pearson correlations (*r*) between all study variables, is provided in Table B1. Comparable to the original study, all variables were significantly and positively associated with one and another at the level of *p* < .01 (two‐tailed).

**TABLE B1:** Bivariate correlations among T1, T2, and change scores related to CPDI infection, symptoms of anxiety, loneliness, and symptoms of depression (*N* = 671)

	M ± SD	Range	1	2	3	4	5	6	7	8	9	10	11	12
1 COVID‐19 Peritraumatic Distress Index (CPDI) T1	35.38 ± 14.12	0–100	—											
2 Anxiety (GAD) T1	3.50 ± 1.65	0–6	.562**	—										
3 Loneliness T1	4.70 ± 1.77	2–8	.358**	.350**	—									
4 Depression (PHQ) T1	3.45 ± 1.64	0–6	.513**	.709**	.412**	—								
5 COVID‐19 Peritraumatic Distress Index (CPDI) T2	31.02 ± 12.78	0–100	.816**	.564**	.291**	.532**	—							
6 Anxiety (GAD) T2	2.65 ± 1.40	0–6	.514**	.637**	.228**	.621**	.698**	—						
7 Loneliness T2	4.50 ± 1.51	2–8	.395**	.254**	.720**	.345**	.417**	.294**	—					
8 Depression (PHQ) T2	2.44 ± 1.39	0–6	.498**	.533**	.356**	.695**	.673**	.778**	.473**	—				
9 Change score COVID‐19 Peritraumatic Distress Index	4.35 ± 8.25	−100–100	.448**	.089*	.163**	.054	−.151**	−.204**	.031	−.188**	—			
10 Change Score Anxiety (GAD)	0.85 ± 1.32	−6–6	.165*	.479**	.197**	.229**	−.028	−.260**	.008	−.152**	.324**	—		
11 Change Score Loneliness	0.20 ± 1.25	−8–8	.029	.189**	.545**	.166**	−.093*	−.033	−.190**	−.068	.193**	.267**	—	
12 Change Score Depression (PHQ)	1.02 ± 1.21	−6–6	.123**	.349**	.154**	.558**	−.056	−.058	−.073	−.210**	.297**	.491**	.306**	—

* *p* < .05 (two‐tailed).

** *p* < .01 (two‐tailed).

#### Longitudinal serial mediation analysis

The results of the validated longitudinal serial mediation analysis for CPDI (IV) on anxiety (M1), loneliness (M2), and on symptoms of depression (DV) controlling for age, gender, educational level, and ICD‐10 diagnosis are shown in Figure B2 and Table B2. Compared to the original study, the results of the present study show that CPDI was not significantly associated with the change score of symptoms of generalized anxiety. However, as in the original study, the change score of symptoms of generalized anxiety was significantly and positively associated with the change score of loneliness. Further, a significant association was found between the change score of the CPDI and the change score of loneliness. Finally, the change score of loneliness significantly predicted the change score of symptoms of depression. The total effect of the independent variable CPDI on the dependent variable symptoms of depression was significant and also remained significant upon the inclusion of the mediator variables in the model.

**TABLE B2:** Hypothesized longitudinal serial mediation model of symptoms of anxiety and loneliness between CPDI and symptoms of depression in *N* = 632 rehabilitation patients (full sample, for the sample of study retainers see Table 2 in the main manuscript)

Pathway	Effect	SE	BootLLCI	BootULCI
Total effect (c)	0.289	0.006	0.029	0.054
Direct effect (c′)	0.131	0.006	0.008	0.030
a1	0.315	0.007	0.036	0.065
a2	0.118	0.007	0.005	0.031
a3	0.226	0.043	0.132	0.300
b1	0.402	0.034	0.293	0.429
b2	0.169	0.035	0.090	0.227
Indirect effects				
Total indirect effects	0.159	0.024	0.112	0.208
Indirect 1	0.127	0.021	0.086	0.171
Indirect 2	0.020	0.009	0.005	0.039
Indirect 3	0.012	0.004	0.005	0.022

*Abbreviations*: Indirect 1, CPDI → symptoms of anxiety → symptoms of depression; Indirect 2, CPDI→ loneliness → symptoms of depression; Indirect 3, CPDI→ symptoms of anxiety → loneliness → symptoms of loneliness. BootLLCI, bootstrapping lower limit confidence interval; BootULCI, bootstrapping upper limit confidence interval; SE, standard error; Effect, standardized regression coefficient.

Symptoms of anxiety as well as loneliness served as independent mediators of the relationship between CPDI and symptoms of depression in the validated analyses. All covariates (gender, age, educational level, and ICD‐10 diagnosis) were not significantly associated with either variable in the serial mediation model. Overall, 28.75% of the variance in symptoms of depression was accounted for.

#### Conclusion

To validate the findings from the original study investigating a serial mediation model only in patients who took part in the survey at both measurement timepoint (pre‐ and post‐rehabilitation), a validation study was performed by assessing the serial mediation model with imputed data for measurement timepoint two. The results of the validation study mimic the results of the original study.

## APPENDIX C

**TABLE C1:** Bivariate correlations among T1, T2, and change scores related to CPDI infection, symptoms of anxiety, loneliness, and symptoms of depression (*N* = 403)

	M ± SD	Range	1	2	3	4	5	6	7	8	9	10	11	12
1 COVID‐19 Peritraumatic Distress Index (CPDI) T1	32.48 ± 13.18	0–100	—											
2 Anxiety (GAD) T1	3.48 ± 1.68	0–6	.580**	—										
3 Loneliness T1	4.57 ± 1.76	2–8	.368**	.381**	—									
4 Depression (PHQ) T1	3.42 ± 1.66	0–6	.508**	.732**	.450**	—								
5 COVID‐19 Peritraumatic Distress Index (CPDI) T2	30.42 ± 13.99	0–100	.700**	.476**	.315**	.449**	—							
6 Anxiety (GAD) T2	2.63 ± 1.66	0–6	.485**	.550**	.303**	.510**	.693**	—						
7 Loneliness T2	4.30 ± 1.70	2–8	.312**	.243**	.607**	.286**	.386**	.379**	—					
8 Depression (PHQ) T2	2.43 ± 1.70	0–6	.478**	.468**	.372**	.581**	.667**	.787**	.491**	—				
9 Change score COVID‐19 Peritraumatic Distress Index	2.06 ± 10.55	−100–100	.321**	.091	.041	.039	−.452**	−.312**	−.123*	−.288**	—			
10 Change Score Anxiety (GAD)	0.85 ± 1.58	−6–6	.108*	.481**	.089	.240**	−.220**	−.467**	−.136**	−.330**	.422**	—		
11 Change Score Loneliness	0.25 ± 1.53	−8–8	.076*	.163**	.474**	.185**	−.076	−.098	−.411**	−.136	.199**	.278**	—	
12 Change Score Depression (PHQ)	0.99 ± 1.54	−6–6	.032	.276**	.080	.442**	−.244**	−.314**	−.228**	−.473**	.362**	.623**	.344**	—

* *p* < .05 (two‐tailed).

** *p* < .01 (two‐tailed).
